# Pericardial anisakiasis: unravelling diagnostic challenges in an unprecedented extra-abdominal manifestation: a case report

**DOI:** 10.1093/ehjcr/ytae093

**Published:** 2024-02-19

**Authors:** Jacopo Giovacchini, Silvia Menale, Valentina Scheggi, Niccolò Marchionni

**Affiliations:** Department of Experimental and Clinical Medicine, University of Florence, Largo Brambilla 3, Florence 50134, Italy; Department of Experimental and Clinical Medicine, University of Florence, Largo Brambilla 3, Florence 50134, Italy; Division of Cardiovascular and Perioperative Medicine, Cardiothoracovascular Department, Careggi University Hospital, Florence, Italy; Department of Experimental and Clinical Medicine, University of Florence, Largo Brambilla 3, Florence 50134, Italy

**Keywords:** Anisakis, Pericardial anisakiasis, Extra-abdominal anisakiasis, Pericarditis, Case report

## Abstract

**Background:**

Anisakis infects humans by consuming contaminated undercooked or raw fish, leading to gastric anisakiasis, gastro-allergic anisakiasis, or asymptomatic contamination. Although larvae usually die when penetrating the gastric tissue, cases of intra- and extra-abdominal spread were described. We report the first probable case of pericardial anisakiasis.

**Case summary:**

A 26-year-old man presented to the emergency department because of progressive lower limb oedema and exertional dyspnoea. Two months prior, he had consumed raw fish without any gastrointestinal symptoms. The echocardiogram reported a circumferential pericardial effusion (‘swinging heart’) and mildly reduced left ventricular ejection fraction (LVEF). He was diagnosed with myopericarditis after a cardiac magnetic resonance. A fluorodeoxyglucose positron emission tomography scan revealed an intense pericardial metabolism. Blood tests exhibited persistent eosinophilia and mild elevation of *Anisakis simplex* IgE—as for past infestation. A pericardial drainage was performed, subsequently, serial echocardiograms revealed a spontaneous recovery of his LVEF. No autoimmune, allergic, or onco-haematologic diseases were identified. Based on a history of feeding with potentially contaminated raw fish and on long-lasting eosinophilia, we suspected a pericardial anisakiasis, despite a low but persistent titre of specific IgE. Albendazole was administered for 21 days, along with colchicine and ibuprofen for 2 months; pericardial effusion resolution and eosinophil normalization occurred two weeks after.

**Discussion:**

We hypothesized that *Anisakis* larvae may have migrated outside the gastrointestinal tract, penetrating the diaphragm and settling in the pericardium, causing pericarditis and pericardial effusion. Clinicians should know that the pericardium may be another extra-abdominal localization of anisakiasis, beyond pleuro-pulmonary involvement.

**Learning points**

- In the differential diagnosis of eosinophilia, clinicians should assess for a wide spectrum of potential underlying aetiologies, including allergic conditions, autoimmune processes, onco-haematologic diseases, or fungal/parasitic infection.
- Eosinophilia with a history of consuming undercooked or raw fish—regardless of the time elapsed—should prompt a search for *Anisakis* even in the absence of gastrointestinal symptoms.
- Extra-gastrointestinal both intra- and extra-abdominal localizations of *Anisakis* are possible.
- Pericardium may be a site of extra-abdominal anisakiasis, with consequent pericarditis and pericardial effusion.

## Introduction

The *Anisakis* nematode inhabits fish and marine mammals and can infect humans if they consume contaminated, undercooked, or raw fish. Anisakiasis is an emerging zoonosis in Europe, with most cases reported in Spain and Italy and less from the Netherlands and Germany;^1^ in particular, Italy is the fourth country with the highest number of documented cases globally, according to a recent review of the literature.^2^ Human contamination may result in gastric anisakiasis, characterized by severe epigastric pain, nausea, and vomiting occurring within hours of ingestion.^3,4^ An IgE-mediated systemic allergic reaction that coexists distinguishes gastro-allergic anisakiasis.^5^ However, infestation may run asymptomatic.^6^ When the larvae penetrate the gastric tissue, they usually die, causing inflammation.^7^ Nevertheless, though rarely, they can spread both inside and outside the abdomen^8^—indeed, cases of pleural localization have been reported.^9,10^ We are describing a probable first case of pericardial anisakiasis.

## Summary figure

**Figure:**
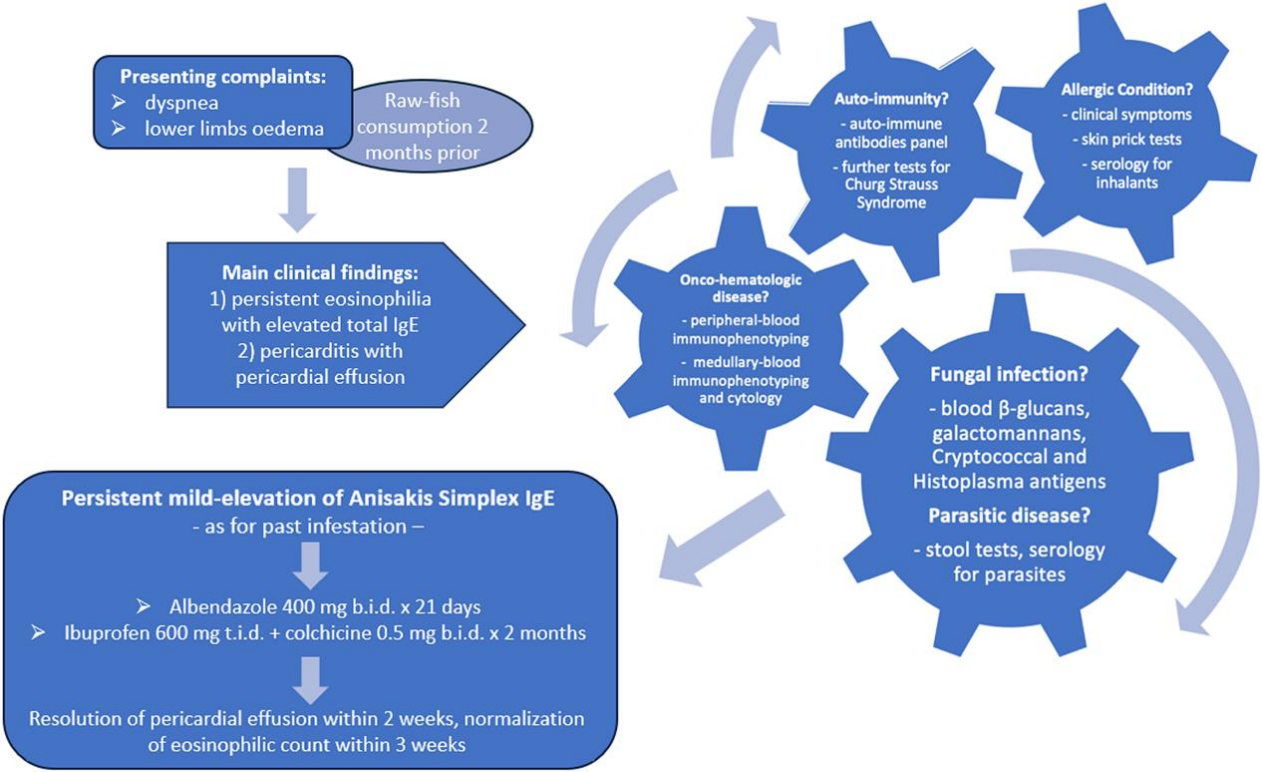


## Case presentation

A 26-year-old man presented to the emergency department with progressive lower limb oedema over the past two weeks, accompanied by shortness of breath during physical activity. He had chronic sinusitis and a family history of coeliac disease but had no known allergies or medication history. Two months prior, he had consumed raw fish without developing any fever or gastrointestinal symptoms, and he had not been in contact with animals recently.

**Figure 1:**
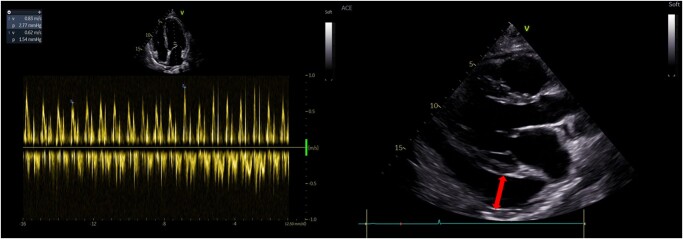
‘Swinging heart’ and 25% breathing variability of mitral pulsed Doppler.

Upon examination, he was stable and apyretic but showed signs of reduced heart sounds and pitting oedema in his lower limbs. Blood tests revealed eosinophilia (980/mm^3^—normal range 0–700/mm^3^), increased total IgE concentration and C-reactive protein levels (12 mg/dL, normal range < 5 mg/dL). His ECG was normal, but an echocardiogram showed a severe pericardial effusion with a ‘swinging heart’, ventricular interdependence, a dilated inferior vena cava, and a 25% breathing variability of mitral pulsed Doppler (*Figure 1*; see Supplementary Material). His left ventricular ejection fraction (LVEF) was reduced to 48%.

Chest and abdomen contrast-enhanced CT scans revealed pleural and pericardial effusion with mediastinal lymphadenopathies measuring up to 15 mm in diameter.

Colchicine 0.5 mg b.i.d. and ibuprofen 600 mg t.i.d. were administered—and continued throughout the hospitalization—to treat suspected pleuro-pericarditis.

After being transferred to the Division of Internal Medicine, he underwent echo-assisted pericardiocentesis and 1150 mL of pericardial serous liquid was drained. Microbiologic tests of pericardial liquid came back negative, and cytology showed normal lymphocytes, macrophages, and mesothelial cells without eosinophils. Over the next few days, the patient’s peripheral oedema subsided and his LVEF recovered, however, the eosinophilia persisted (940–1100/mm^3^) and echocardiography detected a stable residual pericardial effusion (maximum 16 mm posteriorly) throughout the hospital stay.

**Figure 2:**
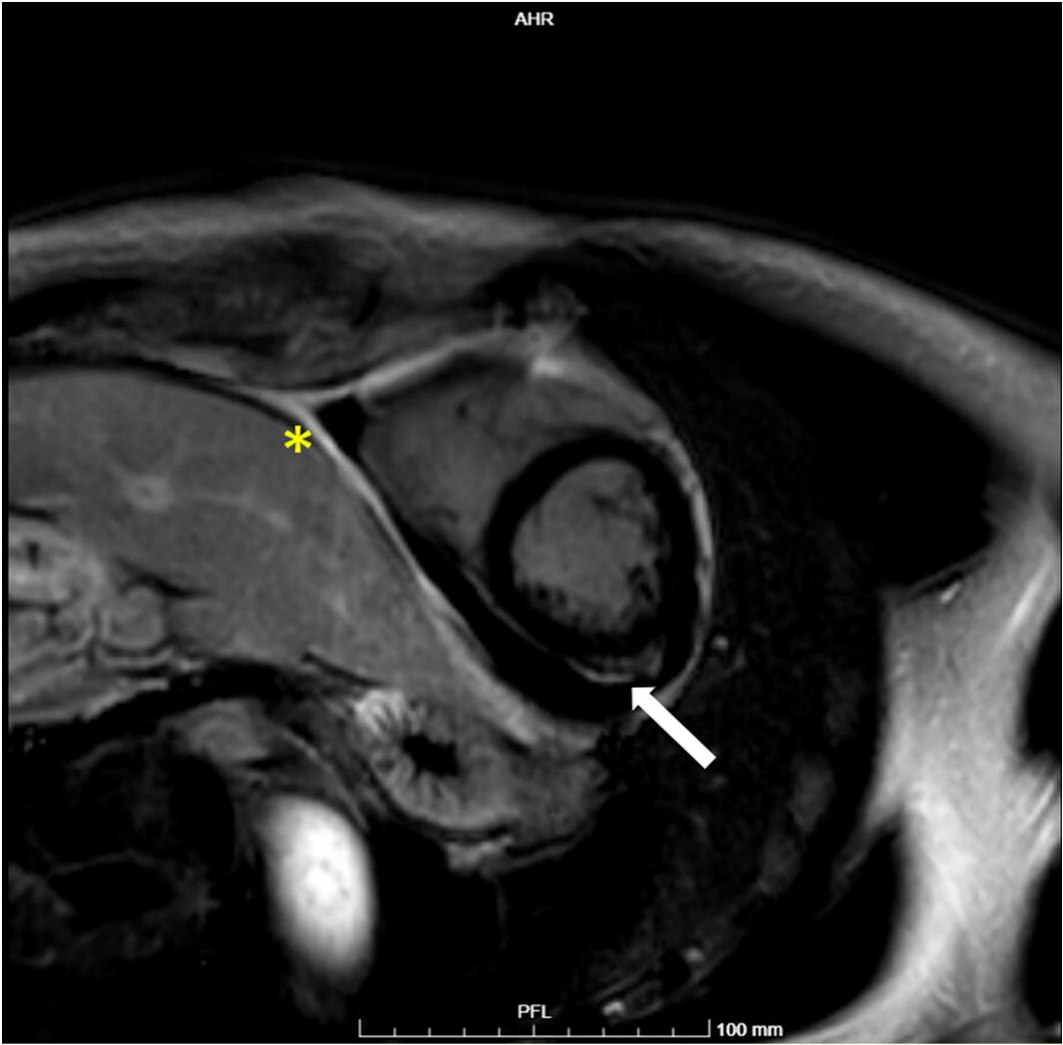
Cardiac MRI, T1-weighted sequence showing pericardial (asterisk) and a subepicardial area of LGE (arrow).

**Figure 3:**
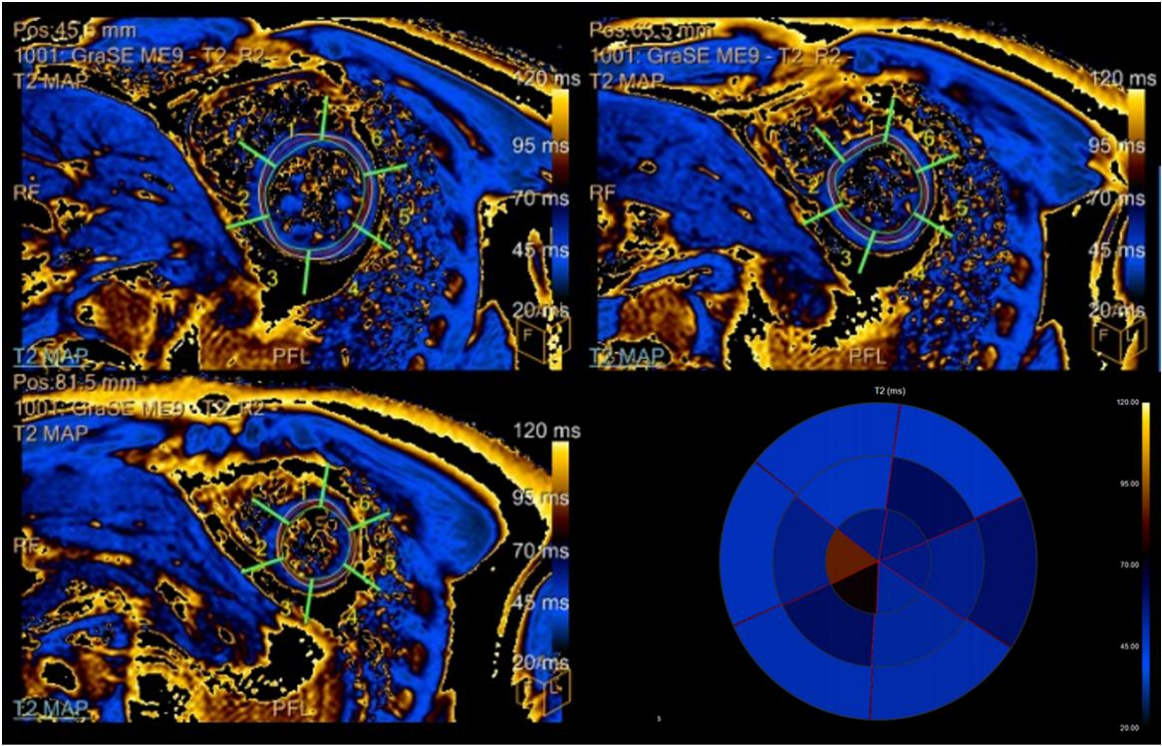
Cardiac MRI, T2-mapping sequence showing myocardial oedema.

**Figure 4:**
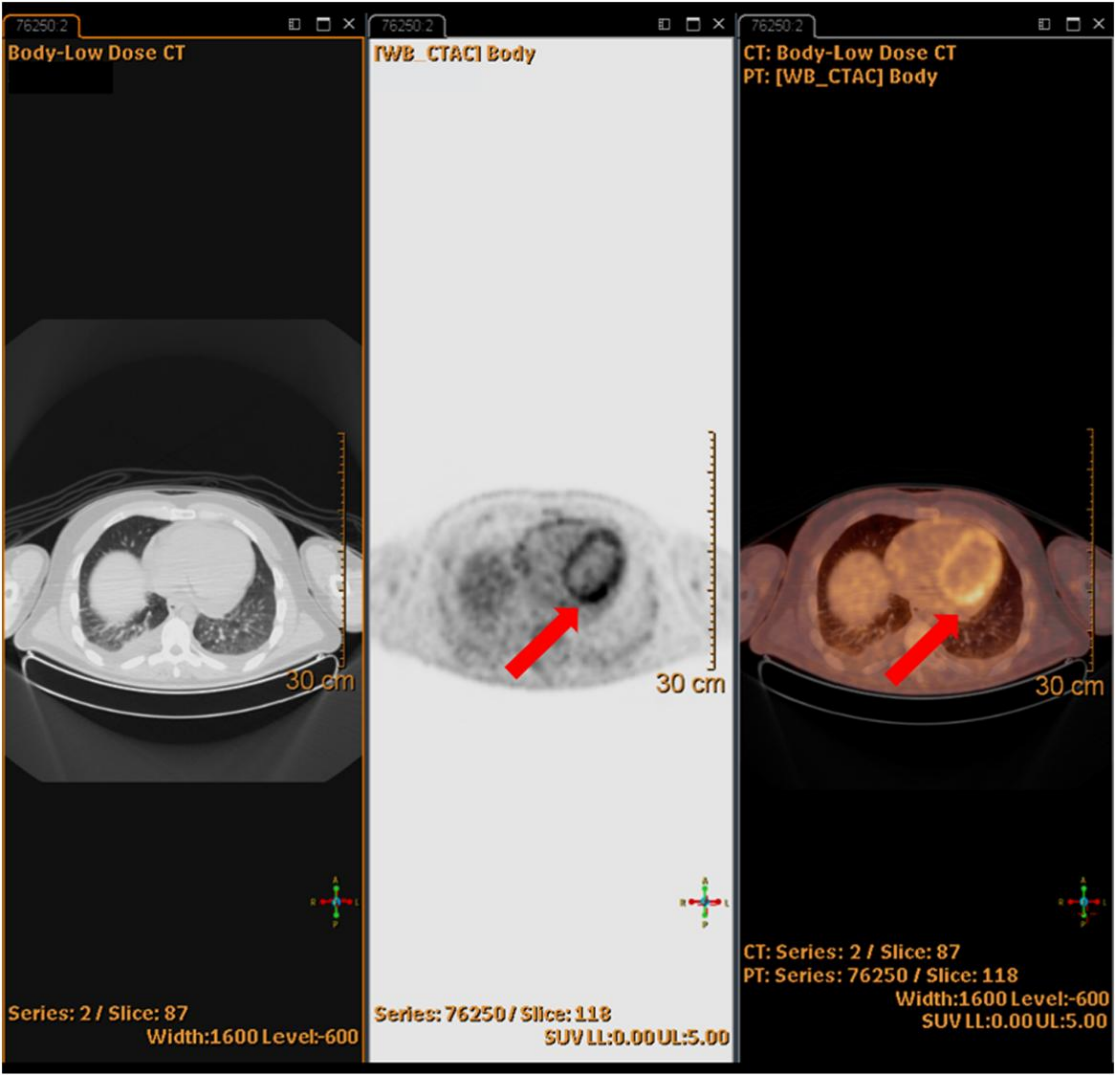
On the left, low-dose body CT scan; in the middle, grey-scale PET-FDG highlighting intense glucose uptake and metabolism in the pericardium as compared to the hepatic dome; on the right, low-dose CT–PET-FDG fused image.

Cardiac magnetic resonance (MRI) reported signs of myopericarditis, including late gadolinium enhancement (LGE) of pericardium and of medium-distal left ventricular subepicardial inferolateral wall (*Figure 2*), with increased T2-mapping (*Figure 3*); LVEF was mildly reduced to 54%. Additionally, the total body fluorodeoxyglucose positron emission tomography (PET-FDG) scan showed notable glucose uptake in the pericardium and mediastinal lymph nodes (*Figure 4*).

An allergic reaction was excluded due to unsuggestive serology for inhalants, negative skin prick tests, and lack of common allergic symptoms.

All serology tests for viral and bacterial aetiologies of pericarditis, as well as blood β-glucans, galactomannans, Cryptococcal and Histoplasma antigens yielded negative results, and stool tests were normal. Serology of *Toxocara canis*, *Strongyloides stercoralis*, *Ascaris lumbricoides*, and *Trichinella spiralis* were also negative. However, *Echinococcus granulosus* IgG and *Anisakis simplex* IgE were mildly elevated [1:64 (<1:32 range for positivity), and 0,33 kUA/L (threshold for positivity at our laboratory > 0,10), respectively), even at repeated tests. Consequently, an abdominal contrast-enhanced MRI was conducted to rule out echinococcosis. On the other hand, due to the low but specific titre presented by the *Anisakis* serology and the atypical clinical presentation, we deemed it necessary to exclude other diseases. Therefore, an onco-haematologic condition was excluded after negative results from peripheral-blood immunophenotyping and medullary-blood immunophenotyping and cytology—which showed an expansion of normal medullary eosinophils. Churg–Strauss syndrome was also investigated due to reported chronic sinusitis, but no auto-antibody titres were elevated, and both rhinoscopy and facial-sinuses CT scan were unsuggestive.

Upon confirmation of stable elevation of *Anisakis*-specific IgE titre and long-lasting eosinophilia, we suspected the patient had consumed contaminated fish, leading to potential pericardial anisakiasis. However, given the time that had passed since the presumed contamination (indeed, the titre of specific IgE was low), we opted not to perform a diagnostic endoscopy. Instead, we prescribed albendazole 400 mg b.i.d. along with colchicine 0.5 mg b.i.d. and ibuprofen 600 mg t.i.d. As the patient remained stable, he was discharged 20 days after admission.

The patient underwent outpatient echocardiography at days 3–7–14–30 from discharge, which demonstrated progressive improvement of pericardial effusion and complete resolution at 2 weeks. Blood tests showed normalization of eosinophilic count and, subsequently, C-reactive protein as well within 3 weeks. Albendazole was stopped after 21 days of treatment; colchicine and ibuprofen were continued and de-escalated within two months of discharge.

## Discussion

The *Anisakis* nematode is found in fish and marine mammals and can infect humans if they consume contaminated, undercooked, or raw fish.^7^ While some individuals may not exhibit symptoms, others may experience epigastric pain, nausea, and vomiting as the larvae burrow into the gastric wall—a condition known as gastric anisakiasis.^3,5,11^ Anaphylaxis may also occur, leading to gastro-allergic anisakiasis.^5^ The larvae usually perish during gastric tissue penetration, potentially causing eosinophilic enterocolitis or focal peritonitis.^7^ However, in exceptional instances, *Anisakis* larvae can survive after penetrating the gastric mucosa, spreading either through the abdominal wall and viscera generating eosinophilic granulomas,^8^ or outside the abdominal cavity, causing pulmonary anisakiasis and pleural effusion.^9,10^ Elevated levels of total and *Anisakis*-specific IgE are often present in affected patients. However, diagnosis can be a challenge, particularly for extra-gastrointestinal anisakiasis,^9^ as identifying the larvae can be difficult due to their degeneration induced by local inflammatory reactions.^12^

To our knowledge, no cases of pericardial anisakiasis have been reported previously. Our patient consumed raw fish two months prior to the onset of congestive heart failure, but did not experience any gastrointestinal symptoms. We hypothesized that *Anisakis* gastrointestinal colonization may have run without causing symptoms, resulting in eosinophilia and elevated total and specific IgE levels. The parasite may have then migrated outside of the gastrointestinal tract, passing through the diaphragm and localizing in the pericardium, with pericarditis taking place over time and resulting in a gradual accumulation of pericardial effusion. Indeed, the symptoms appeared almost two months after the presumed contamination. It is noteworthy that patients with extra-abdominal anisakiasis have also been reported to lack abdominal manifestations.^9,10^ Although our patient did not exhibit typical ECG changes or pericardial pain, both PET-FDG and cardiac MRI revealed inflammation in the pericardium. Testing of pericardial fluid did not detect *Anisakis* larvae, which is consistent with previous cases of pleural anisakiasis where the pathogen was not identified through microbiologic testing.^12^

Our case has some limitations. Firstly, we did not perform a biopsy of the pericardium to confirm our diagnosis and check for traces of larvae. However, serological tests are reliable in cases of chronic anisakiasis, as specific IgE levels remain elevated even after two months from infestation.^11,13^ Secondly, we did not find eosinophils within pericardial fluid, while they were reported in pleural cytologic tests of patients with pleuro-pulmonary anisakiasis.^9^ In addition, the *Anisakis*-specific IgE titre in our patient was low and unsuggestive of an acute infestation, as stated elsewhere.^14^ However, these findings could be consistent with a past contamination. The rapid resolution of pericardial effusion and normalization of eosinophil count after anti-parasite treatment indirectly confirmed our diagnosis. It’s worth noting that in a case of pleuro-pulmonary anisakiasis where anti-parasitic treatment has not been carried out, the eosinophil count normalized after 7 months.^9^ Thirdly, we did not perform a cardiac MRI to check for LGE and T2-mapping restoration after anti-parasite treatment, instead the patient underwent a meticulous echocardiographic follow-up, demonstrating the resolution of pericardial effusion.

## Conclusions

Anisakiasis is an emerging zoonosis in Europe. Diagnosis can be a challenge, especially when extra-gastrointestinal localizations occur. Clinicians should be aware that the pericardium may be another extra-abdominal localization of anisakiasis, beyond pleuro-pulmonary involvement.

## Supplementary Material

ytae093_Supplementary_Data

## Data Availability

Data and material are available on reasonable request from the author.

## Lead author biography

Jacopo Giovacchini is a Cardiology Resident based in Florence, Italy. With a keen interest in arrhythmology and intensive care, he is deeply passionate about all aspects of clinical cardiology, from prevention to diagnosis and treatment.

## Supplementary material

**Consent:** The authors confirm that a written informed consent for the publication of this case report was obtained from the patient, in line with the Committee on Publication Ethics (COPE) guidelines.
